# Response: Enhancing the validity and applicability of study for health-related quality of life in patients with conditions affecting the hand

**DOI:** 10.1093/bjs/znae218

**Published:** 2024-09-02

**Authors:** Luke Geoghegan, Conrad J Harrison, Jeremy N Rodrigues

**Affiliations:** Department of Surgery and Cancer, Imperial College London, London, UK; Nuffield Department of Orthopaedics, Rheumatology and Musculoskeletal Sciences, University of Oxford, Oxford, UK; Department of Plastic and Reconstructive Surgery, Stoke Mandeville Hospital, Aylesbury, UK; Warwick Clinical Trials Unit, University of Warwick, Coventry, UK

Dear Editor

We thank Chen *et al.* for their correspondence and criticism of our work. We acknowledge the importance of publication bias on interpretation of our findings; however, statistical tests for publication bias must be used with caution. Egger’s test can be used to indicate funnel plot asymmetry, although it fails to account for between-study heterogeneity. Differences in baseline condition severity, co-morbidities, functional status, employment and even handedness may all impact on health utility estimates even when the same valuation technique is used. We highlight between-study heterogeneity as an inherent limitation of pooled utility estimates and advise that estimates be used by analysts with caution. The trim-and-fill method is an impractical solution as it assumes publication bias as the sole reason for funnel plot asymmetry, which in the present meta-analysis is an unrealistic assumption^1^.

We agree that condition severity, valuation techniques and respondent characteristics are all likely to account for between-study heterogeneity and have attempted to account for these in our meta-analysis. Lack of primary data and non-standardized condition severity reporting among primary studies precludes meaningful substratification by all factors likely to influence health utility scores.

The EQ-5D has been shown to have poor responsiveness in hand conditions and its use in cost–utility analyses of interventions for hand conditions is indeed questionable. The authors state that incorporation of condition-specific preference-based measures could prove more ‘accurate’, although, to date, a value set for hand-specific patient-reported outcome measures is yet to be derived.

Finally, our search was not geographically restricted, and predominance of Western studies likely reflects global publication trends.
